# Providing successful faculty development to graduate medical education program directors

**DOI:** 10.5116/ijme.59a9.73cc

**Published:** 2017-09-11

**Authors:** Alisa Nagler, Kathryn M. Andolsek, Mariah Rudd, Catherine M. Kuhn

**Affiliations:** 1 Division of Education, American College of Surgeons, United States of America; 2 Department of Community and Family Medicine, Duke University School of Medicine, United States of America; 3 Office of Continuing Professional Development, Virginia Tech Carilion School of Medicine, United States of America; 4 Department of Anesthesiology, Duke University School of Medicine, United States of America

**Keywords:** Faculty development, graduate medical education, program director

## Introduction

Graduate Medical Education (GME) is the portion of the medical education continuum that spans the period following graduation from medical school to independent practice.  In the United States, successful completion of GME is essential for board certification in one of over 140 recognized specialties. The Accreditation Council for Graduate Medical Education (ACGME) is the accreditor for over 10,000 programs. Each program is required to have a single physician program director responsible for the program’s administrative and educational oversight. Virtually all program directors remain clinically and academically productive. 

Over the last 17 years, the ACGME has transitioned its accreditation emphasis to promote competency based education. Furthermore, the ACGME requires programs to provide faculty development in teaching and assessment for their faculty and to evaluate the program's performance in providing it annually.^1^ Some program directors, selected for their interest in teaching and advocacy for residents, have never been formally trained as educators or as educational leaders.^2^ Previous studies have reported that program directors feel poorly prepared to meet their educational and accreditation challenges. Among their top concerns are a lack of expertise in curriculum development and evaluation, and a lack of understanding of ACGME competencies and their assessment.^3^

Furthermore, faculty attrition has been linked to inadequate faculty development.^4^ In response, many program directors turn to external sources of professional development outside of their individual programs.^5^ Hafler and colleagues highlight the importance of faculty development efforts in providing knowledge and skills while facilitating a supportive community in which continual improvement of teaching and assessment becomes the norm.^6^

We used situational learning theory and best practices from communities of practice, to develop implement, and evaluate an institutional faculty development program for over 100 GME program directors of both ACGME accredited and internally sponsored programs. The purpose of this Perspective is to present our eight-year experience.

The faculty development program

Workshop topics were identified through a literature review, an online survey of program directors soliciting the major gaps in their educational competence and confidence, an analysis of the citations programs had received for deficiencies from the ACGME and a review of the major changes in ACGME requirements.  Each topic was developed into an hour-long workshop.  Six to 8 topics were offered each spring and fall.  These topics were each presented three different times and days during a single week to maximize program director attendance.  Sessions were recorded using the technology available through the institution. Presentation materials, including practical tools and resources, were compiled into a toolkit and distributed at each workshop for program directors to use within their own programs. These materials were also posted to a password protected website.  A certificate of exemplary attendance was sent to the Departmental Chairs of participants who attended 10 or more sessions over the course of an academic year. Evaluations were designed to measure participant satisfaction and elicit feedback for improving future workshops and identify timely topics. We used Kirkpatrick’s four levels of evaluation to evaluate the program.

Over the course of eight academic years, from fall 2007 through fall 2014, 97 different workshops were presented to over eleven hundred participants. An archived library was created by uploading recordings of the workshops, presentation slides and resource toolkits. The workshops were categorized by themes which ranged from “ACGME Competencies” to “Technology & Social Media in Teaching.” Workshops were co-taught by internal experts from across the institution.

There were eleven hundred and twenty session attendees, consisting of two hundred and four unique individuals who attended at least one session. Forty percent of program directors attended sessions each year and represented twelve of the then thirteen clinical departments. Sixty-eight percent planned to use the electronic tool kits. Participants regularly attended multiple different sessions based on their availability and their specific needs for the content being offered. Session attendance fluctuated with the time of day and year. Attendance was best for sessions held at noon (546 attendees).

Four hundred and seventy evaluations (42%) were returned over the course of the series. The aggregate response results demonstrated participants found the logistics, content and networking opportunities provided by the sessions to be “extremely helpful”.  The cost was modest.

## Conclusions

We demonstrated a high rate of participation and satisfaction with a voluntary institutional faculty development activity for program directors and individuals with major GME roles. Workshops covered critical content identified by participants and through gaps noted by the accrediting body. The format was convenient, practical, and participative. Materials were provided that could be put to immediate use. Because there was no one day and time that accommodated the majority of attendees, we offered both live and enduring formats. Following Kirkpatrick’s four levels of evaluation, the evaluation data demonstrate learner satisfaction (level 1).  While more difficult to measure, the authors believe workshop participants learned (level 2) and changed their behavior (level 3), potentially leading to positive results (level 4).^7^ Strategies for recruitment, remediation for sub-optimally performing residents, program director twice yearly reviews with residents, and peer evaluations were frequently adopted (level 3 - changed behavior).

We believe there was increased program director satisfaction, greater networking, enhanced program quality and improved perception of the value added of the institutional GME office beyond administrative functions. The success and feasibility of this model of faculty development helped lead to an institution-wide monthly Medical Education Grand Rounds, a newly implemented Faculty Teaching Academy, and greater collaboration supporting medical education research and scholarship.

We believe the networking opportunities provided by these workshops helped create a community of educators committed to continual improvement of themselves, their programs, one another, and the institution. We believe other medical educators can easily adapt our model of faculty development to their own institutions.

### Conflict of Interest

The authors declare that they have no conflict of interest.
